# Angiopoietin-2 as a prognostic biomarker in septic adult patients: a systemic review and meta-analysis

**DOI:** 10.1186/s13613-024-01393-0

**Published:** 2024-11-10

**Authors:** Mengke Zhuo, Sifeng Fu, Yawen Chi, Xinghua Li, Sirui Li, Xiaochun Ma, Xu Li

**Affiliations:** 1 Department of Critical Care Medicine, The First Affiliated Hospital of China Medical University, North Nanjing Street 155, Shenyang, Liaoning Province 110001 China; 2 Department of Respiratory and Critical Care Medicine, The First Affiliated Hospital of Guangzhou Medical University, Guangzhou Institute of Respiratory Health, State Key Laboratory of Respiratory Diseases, Guangzhou, 510120 China

**Keywords:** Angiopoietin-2, Sepsis, Biomarkers, Mortality, Meta-analysis

## Abstract

**Background:**

The impairment of endothelial function represents a key pathophysiological mechanism in the development of sepsis. This research aimed to evaluate the prognostic significance of angiopoietin-2 (Ang-2), an endothelial biomarker, in predicting mortality in sepsis patients.

**Methods:**

Chinese and English studies were systematically retrieved in PubMed, Cochrane Library, Embase, WanFang, CNKI, CQVIP, and CBM databases up to July 16, 2023. We conducted a study selection established upon predefined inclusion and exclusion criteria and used the Newcastle-Ottawa scale (NOS) to assess its quality. We extracted available data from the included studies for data analysis.

**Results:**

The final inclusion comprised 33 studies with 4703 participants. According to the NOS, one study was of medium quality, while the rest were of high quality. In comparison to survivors, the levels of Ang-2 in non-survivors were markedly elevated [standardized mean difference (SMD) = 1.08, 95% confidence intervals (CI) 0.68–1.49, *P* < 0.001], and the same results were also observed in the subgroup that met sepsis 3.0 diagnosis criteria (SMD = 0.63, 95% CI 0.11–1.14, *P* = 0.017). The results comparing Ang-2 levels between non-survivors and survivors were independent of duration of follow-up, sample sources, type of study, and region. Ang-2 was a risk factor for mortality [odds ratios (OR) = 1.16, 95% CI 1.09–1.23, *P* < 0.001]. Ang-2 was demonstrated to be able to predict mortality in septic adult patients [area under the curve (AUC) = 0.76, 95% CI 0.70–0.82, *P* < 0.001].

**Conclusions:**

Ang-2 level was positively correlated with risk of death in sepsis patients. Ang-2 might be a useful and valuable biomarker for predicting mortality in septic adult patients.

**Supplementary Information:**

The online version contains supplementary material available at 10.1186/s13613-024-01393-0.

## Introduction

Sepsis is fatal organ dysfunction due to an uncontrolled immune response to infection [1]. Although the Surviving Sepsis Campaign (SSC) guideline has acknowledged the potential significance of biomarkers in the treatment and prediction of the prognosis of sepsis, it fails to provide recommendations for the use of any biomarker for predicting sepsis prognosis [2]. Due to the intricate interplay of procoagulant, pro-inflammatory, and anti-inflammatory mechanisms that underlie the pathophysiology, identifying the ideal biomarker for sepsis is a challenge. Endothelial cells, which line the luminal surface of all blood vessels, serve as the first barrier separating blood from organs. Therefore, endothelial cells are the first sites to be invaded by pathogens. Endothelial activation or dysfunction is recognized as one of the main pathophysiological mechanisms and directly contributes to the morbidity and mortality of sepsis [3, 4].

Angiopoietin-2 (Ang-2), a growth factor secreted by endothelial cells in response to noxious or inflammatory stimuli, is associated with increased vascular permeability. It has gained much attention as one of the key regulators of microvascular endothelial function over the past two decades [5, 6]. Ang-2 has dual potential in clinical practice: as a prognostic biomarker and as a therapeutic target for conditions characterized by vascular leakage [7, 8]. Wollborn et al. conducted research on patients after cardiac surgery and discovered that Ang-2 was associated with capillary leak and could predict complications after cardiac surgery [7]. In another study, Ang-2 levels were higher in patients with Capillary Leak Syndrome (CLS) compared to those without CLS at various time points, and 30-day mortality was higher in CLS patients [8].

Ang-2 has been extensively studied in the context of sepsis. The function of Ang-2 in sepsis is intricate, exhibiting both beneficial and detrimental effects. In addition to its established role in angiogenesis, it also helps optimize the control of local infections, such as recruiting inflammatory cells and inducing appropriate permeability to facilitate migration [9]. The effects of Ang-2 may become harmful whenever this initial local response transforms into a systemic condition characterized by a widespread and overwhelming immune response [10]. A meta-analysis by Pregernig et al. [11] revealed that in adult sepsis patients, non-survivors exhibited elevated levels of Ang-2 compared to survivors. However, with the updated definition of sepsis, the conclusions of relevant studies were inconsistent [12–14]. Therefore, this meta-analysis aims to assess the potential prognostic significance of Ang-2 in septic adult patients in a larger population and under sepsis 3.0 diagnostic criteria.

## Methods

This research was executed following the protocols outlined in the 2020 Preferred Reporting Items for Systematic Reviews and Meta-Analyses (PRISMA) Statement [15] (Supplementary Material 1).

### Search strategy

PubMed, Cochrane Library, Embase, WanFang, CNKI, CQVIP, and CBM databases were last retrieved on 07/16/2023, and the subject terms were from PubMed’s Medical Subject Headings (MeSH) and SinoMed’s Chinese Medical Subject Headings (CMeSH). For example, in PubMed, the operators “AND” and “OR” are used to search for a combination of “Sepsis” [MeSH], “Septic shock” [MeSH], “Angiopoietin-2” [MeSH], “Prognosis” [MeSH], and the corresponding keywords. The search strategy underwent slight modification to meet the specific requirements of each individual database. The specific details of each database were provided in Supplementary Material 2. Furthermore, to identify potentially relevant articles, we thoroughly examined the bibliographies of the included articles.

### Study inclusion and exclusion criteria

Two authors (MKZ, YWC) independently screened the abstracts and titles based on the following inclusion criteria and then retrieved and reviewed the full texts. Any discrepancies were resolved by consensus. Inclusion criteria: (1) observational study, and the language was limited to English or Chinese; (2) the study subjects were aged 18 years or older and had sepsis, including severe sepsis and septic shock; (3) Ang-2 as an indicator for predicting mortality in sepsis; (4) survival outcomes; (5) one of the following types of data could be obtained directly or indirectly from the study: mean Ang-2 concentrations in both the survivor and non-survivor cohorts, the hazard ratio (HR) or OR with its 95% CI to analyze the correlation between Ang-2 and mortality, and the AUC with its 95% CI to predict mortality. Exclusion criteria: (1) in vitro or animal studies; (2) non-original studies, such as reviews, conference abstracts, comments, letters to editors, etc.; (3) studies with duplicate subjects. For studies with replicate observations, we included more recent studies or studies that provided additional data.

### Quality assessment

The quality measurement was carried out independently by two researchers (XHL, MKZ) using the NOS, and any inconsistencies were addressed in collaboration with an additional researcher (SRL). The NOS comprises three components: selection of the study population, inter-group comparability, and measurement of outcomes. The detailed criteria and their corresponding scores are outlined as mentioned below: the representation of the exposed cohort (1); selection of the unexposed cohort (1); identification of exposure variables (1); demonstration that absence of the anticipated outcomes at baseline (1); comparability between groups established upon research design or statistical analysis (2); outcome assessments were accurate and unbiased (1); adequate follow-up to achieve outcomes (1); cohort follow-up was adequate (1). Every “yes” answer was scored. According to the overall score, research with a score of 7–9 was categorized as high quality, 4–6 as medium quality, and 0–3 as low quality.

### Data extraction

Two authors (MKZ, SFF) independently extracted the following data from the published studies and corresponding additional materials, subsequently recording them in Microsoft Excel. When there were discrepancies in the data, the two authors collaborated to ensure consistency. The data included the following: (1) study information: first author, year of publication, region, and study type; (2) demographic and clinical information: study background, setting, study size, diagnostic criteria for sepsis, disease severity, infection site, whether patients with immunodeficiency or a history of radiotherapy and chemotherapy for cancer were excluded, treatments, and follow-up duration. (3) Ang-2 testing: the time point and method; (4) results: the mean Ang-2 levels in the cohorts of survivors versus non-survivors, the HR or OR with its 95% CI for analyzing the correlation between Ang-2 and mortality, the AUC and its 95% CI for predicting mortality, and the mean Ang-2 levels of patients in the sepsis cohort across different disease severity groups. When extracting Ang-2 levels from two groups, we recorded the data at the earliest time point if there were multiple points of data. For the analysis of OR, multivariate adjusted values were extracted and analyzed. If the values of multivariate analysis are not available, the values of univariate analysis are utilized. For studies that did not report the required data for the analyses, we asked the authors for the necessary data.

### Statistical analysis

All statistical analyses were performed utilizing Stata 14.0. We mainly assessed the relationship between Ang-2 measurements and mortality. Outcomes were assessed using SMD, OR, AUC, and their respective 95% CI. The mean and standard deviation (SD) values were essential for calculating SMD. For continuous variables reported as median (interquartile range) or median (minimum to maximum value), the mean with SD was estimated using the methods reported by McGrath et al. [16]. If studies reported standard errors of the mean (SEM) instead of SD, the SD was computed using the formula provided by the Cochrane Collaboration: SD = SEM * √(sample size) [17]. For effect sizes [(OR or HR) and AUC], the pooled effect size and 95% CI were calculated. If a study provided only HR, it was included in the analysis as equivalent to the OR value. In order to assess heterogeneity, the *I*^*2*^ index was calculated, when the *I*^*2*^ value is greater than 50%, heterogeneity is considered to be high. For the results showing significant heterogeneity and including more than 10 studies, we conducted subgroup analysis and sensitivity analysis in order to identify the origin of heterogeneity and evaluate the robustness of the outcomes. If the source of heterogeneity could not be identified, a random-effect model was applied to data analysis. When *I*^*2*^ ≤ 50%, the heterogeneity was deemed acceptable and a fixed-effect model was adopted. Funnel plots and Egger’s linear regression method were conducted to evaluate the publication bias in the included research. If necessary, the stability of the results was further assessed by the trim-and-fill method.

## Results

### Study selection and characteristics of included studies

**Fig. 1:**
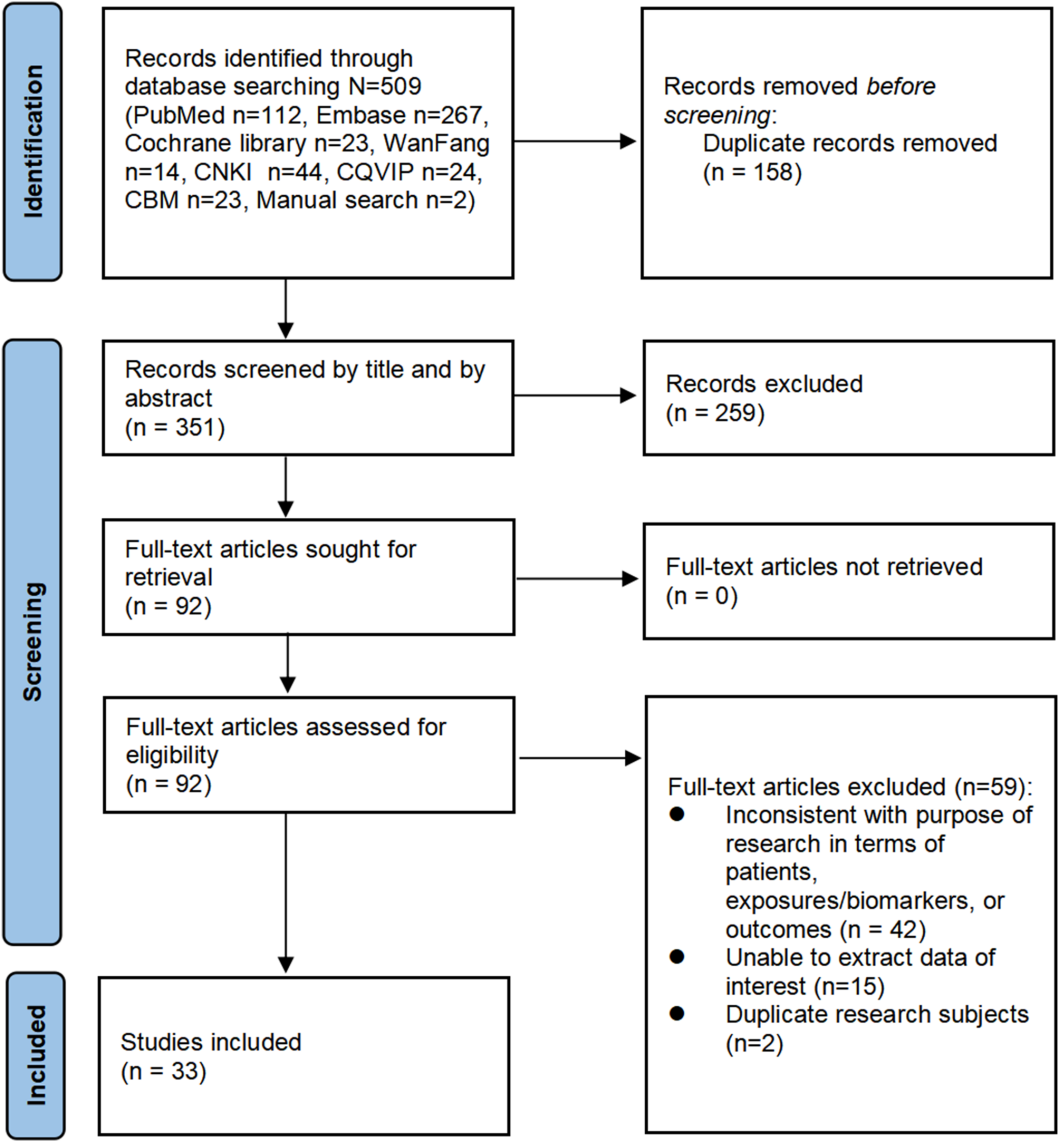
Preferred reporting items for systematic reviews and meta-analyses (PRISMA) flow chart

A total of 509 articles were obtained through electronic and manual searches, with a final inclusion of 33 articles for the meta-analysis, including 23 English articles and 10 Chinese literatures. The process of research selection is shown in Fig. 1.

The basic features of the studies included are outlined in Table 1 and Supplementary Material 3. Of the 33 studies included, 16 used sepsis 3.0 diagnostic criteria, two studies did not specify diagnostic criteria. 17 studies measured plasma Ang-2 levels, while 16 studies measured serum Ang-2 levels. 25 studies described infection sites, mainly in the lungs, abdomen, and urinary system. Excluding two studies that did not specify the follow-up duration, 18 studies provided 28-day mortality, seven studies provided in-hospital mortality, five studies observed 30-day mortality, and the remaining one reported 90-day mortality. The overall methodological assessment of all included studies as evaluated by the NOS indicated a moderate to high level of quality (Supplementary Material 4).

**Table 1:** Characteristics of all included studies

Author	Year	*N*	Region	Design	Definition	Severity	Source	NOS Score
Anderson [18]	2019	400	Non-Asia	MC, PC	Sepsis 3.0, sepsis 2.0	Sepsis, severe sepsis, septic shock	Plasma	8
Belli [13]	2022	35	Non-Asia	SC, PC	Sepsis 3.0	Septic shock	Plasma	9
Beurskens [12]	2020	21	Non-Asia	SC, PC	Sepsis 3.0	Sepsis, septic shock	Plasma	8
Davis [19]	2010	83	Non-Asia	SC, PC	Sepsis 1.0	Sepsis, severe sepsis	Plasma	7
Fang [20]	2015	440	Asia	SC, PC	Sepsis 2.0	SIRS, sepsis, severe sepsis, septic shock	Serum	7
Inkinen [21]	2019	619	Non-Asia	MC, RC	Sepsis 1.0	Sepsis, septic shock	Plasma	9
Karamouzos [14]	2021	128	Non-Asia	MC, RC	Sepsis 3.0	Sepsis	Plasma	9
Kazune [22]	2019	89	Non-Asia	MC, PC	Sepsis 3.0	Septic shock	Plasma	8
Kranidioti [23]	2009	90	Non-Asia	MC, PC	Sepsis 1.0	Sepsis, severe sepsis, septic shock	Serum	7
Palud [24]	2015	20	Non-Asia	SC, PC	Sepsis 2.0	Septic shock	Plasma	7
Ricciuto [25]	2011	70	Non-Asia	MC, PC	Modified sepsis 1.0	Severe sepsis	Plasma	8
Seol [26]	2020	145	Asia	SC, RC	Sepsis 3.0	Sepsis, septic shock	Plasma	8
Sexton [27]	2020	86	Non-Asia	SC, PC	Sepsis 3.0	Sepsis	Plasma	8
Siner [28]	2009	46	Non-Asia	SC, PC	Sepsis 1.0	Sepsis, severe sepsis, septic shock	Serum	7
Walborn [29]	2020	103	Non-Asia	MC, RC	Sepsis 1.0, sepsis 2.0	Sepsis	Plasma	7
Higgins [30]	2018	166	Non-Asia	MC, RC	Sepsis 1.0	Sepsis, severe sepsis, septic shock	Plasma	7
Lin [31]	2015	96	Asia	SC, PC	Sepsis 1.0	Severe sepsis	Plasma	8
Kümpers [32]	2009	21	Non-Asia	SC, PC	Not mention	Sepsis	Serum	6
Ma [33]	2020	41	Asia	SC, RC	Sepsis 3.0	Sepsis with ARDS	Plasma	7
Rosenberger [34]	2023	757	Non-Asia	SC, RC	Sepsis 1.0	Sepsis	Plasma	8
Statz [35]	2018	101	Non-Asia	MC, PC	Sepsis 3.0	Sepsis, septic shock	Plasma	8
Villar [36]	2021	232	Non-Asia	MC, PC	Sepsis 3.0	Sepsis	Serum	7
Parikh [37]	2006	22	Non-Asia	SC, PC	Sepsis 2.0	Sepsis, severe sepsis, septic shock	Serum	7
Chen [38]	2020	36	Asia	SC, PC	Sepsis 3.0	Sepsis, septic shock	Serum	7
Guan [39]	2021	110	Asia	SC, PC	Sepsis 3.0	Septic shock	Serum	7
Lei [40]	2022	45	Asia	SC, PC	Sepsis 3.0	Sepsis with ARDS	Serum	7
Li [41]	2018	67	Asia	SC, PC	Sepsis 3.0	Sepsis with ARDS	Serum	9
Liang [42]	2021	112	Asia	SC, RC	Not mention	Septic shock	Serum	7
Sun [43]	2022	103	Asia	SC, PC	Sepsis 3.0	Sepsis with ARDS	Serum	9
Wang [44]	2021	180	Asia	SC, PC	Sepsis 1.0	Sepsis, severe sepsis	Serum	8
Wen [45]	2021	79	Asia	SC, PC	Sepsis 3.0	Sepsis with ARDS	Serum	8
Wu [46]	2022	75	Asia	SC, PC	Sepsis 3.0	Sepsis	Serum	7
Zhang [47]	2022	85	Asia	SC, PC	Sepsis 1.0	Sepsis with ARDS	Serum	8

*SC* single center, *MC* multiple centers, *PC* prospective cohort study, *RC* retrospective cohort study, *SIRS s*ystemic inflammatory response syndrome, *ARDS a*cute respiratory distress syndrome

## Comparison of Ang-2 levels in survivors versus non-survivors

**Fig. 2:**
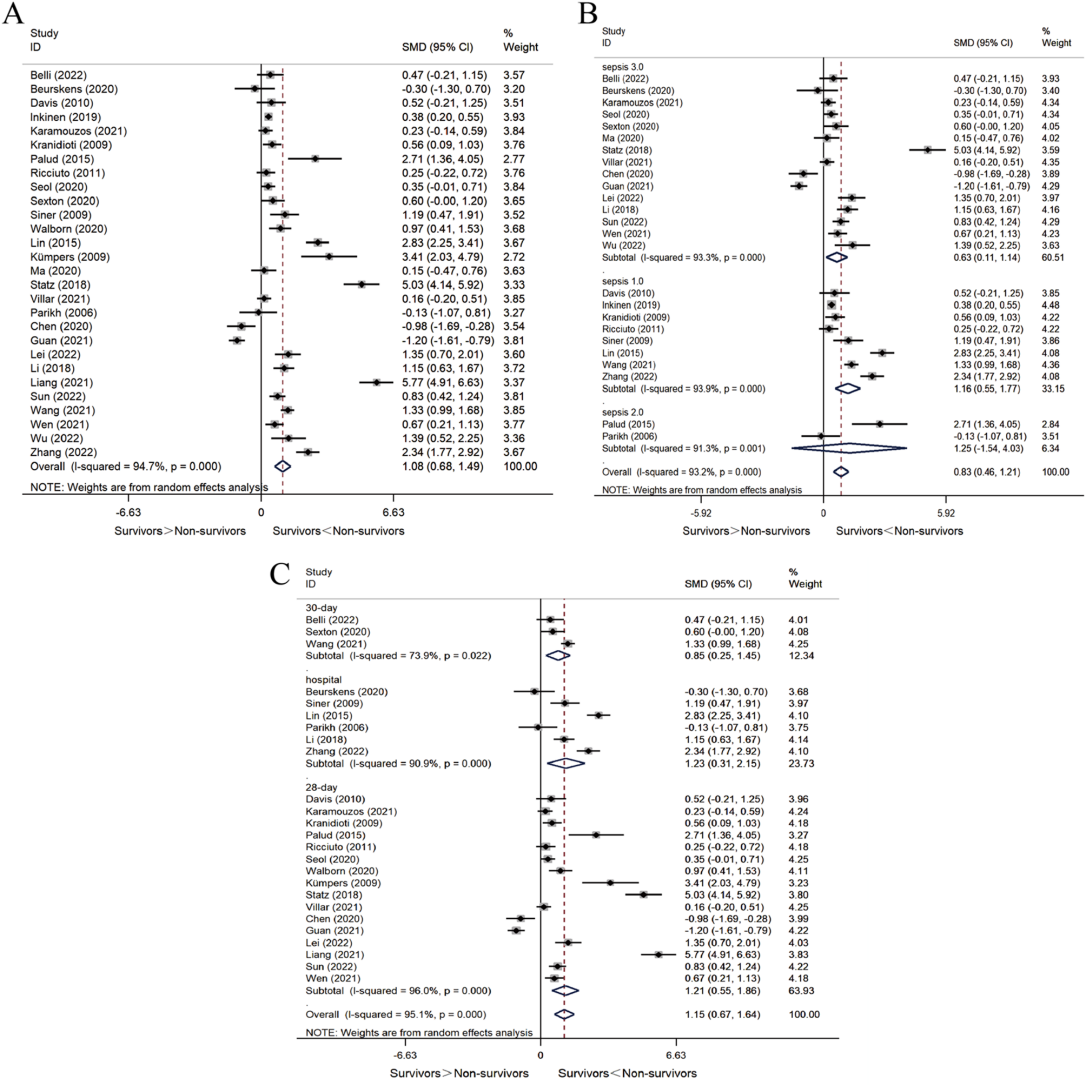
Forest plots of SMD in Ang-2 biomarker measurements in survivors vs. non-survivors. When SMD exceeds 0, it implies that Ang-2 levels are higher in non-survivors compared to survivors. **A** SMD of Ang-2 levels in survivors vs. non-survivors. **B** SMD of Ang-2 levels in survivors vs. non-survivors (subgroup analysis according to follow-up duration). **C** SMD of Ang-2 levels in survivors vs. non-survivors (subgroup analysis according to sepsis diagnostic criteria)

A total of 28 studies compared Ang-2 concentrations between survivors and non-survivors. Considering the significant heterogeneity in the results (*I*^*2*^ = 94.7%, *P* < 0.001), we employed a random-effect model and adopted SMD as the metric for evaluating the effects. The results showed that Ang-2 levels were significantly higher in non-survivors compared to survivors (SMD = 1.08, 95% CI 0.68–1.49, *P* < 0.001) (Fig. 2A). We conducted subgroup analyses to assess the effects of diagnostic criteria, follow-up duration, sample sources, type of study, and region.

In 15 studies using sepsis 3.0 diagnostic criteria, Ang-2 levels were also significantly higher in non-survivors than in survivors (SMD = 0.63, 95% CI 0.11–1.14, *P* = 0.017). A high *I*^*2*^ value of 93.3% (*P* < 0.001) indicated significant statistical heterogeneity in this subgroup (Fig. 2C). In eight studies that used sepsis 1.0 diagnostic criteria, Ang-2 levels were also higher in the non-survivors than in survivors (SMD = 1.16, 95% CI 0.55–1.77, *P* < 0.001). A high *I*^*2*^ value of 93.9% (*P* < 0.001) indicated significant statistical heterogeneity in this subgroup (Fig. 2C). In two studies using sepsis 2.0 diagnostic criteria, there was no statistically significant difference in Ang-2 levels between survivors and non-survivors (SMD = 1.25, 95% CI -1.54-4.03, *P* = 0.380). A high *I*^*2*^ value of 91.3% (*P* < 0.001) indicated significant statistical heterogeneity in this subgroup (Fig. 2C).

Three studies provided 30-day mortality, six studies provided in-hospital mortality, and 16 studies provided 28-day mortality. Subgroup analysis suggested that follow-up duration was not a source of heterogeneity (30-day mortality: *I*^*2*^ = 73.9%, *P* = 0.022; in-hospital mortality: *I*^*2*^ = 90.9%, *P* < 0.001; 28-day mortality: *I*^*2*^ = 96.0%, *P* < 0.001). The pooled results of subgroup analysis were consistent with the overall results, showing that non-survivors presented higher levels of Ang-2 than survivors (SMD = 1.15, 95% CI 0.67–1.64, *P* < 0.001) (Fig. 2B).

13 studies quantified plasma Ang-2 levels, while 15 studies measured serum Ang-2 levels. Subgroup analysis demonstrated that the sample sources did not contribute to the observed heterogeneity (plasma: *I*^*2*^ = 93.4%, *P* < 0.001; serum: *I*^*2*^ = 95.6%, *P* < 0.001). Consistent with the overall findings, the pooled results of subgroup analyses showed that non-survivors presented higher Ang-2 levels than survivors (Supplementary Material 5).

22 studies were prospective observational studies, while six studies were retrospective. Subgroup analysis revealed that the study type was not a contributor to heterogeneity (prospective cohort study: *I*^*2*^ = 93.9%, *P* < 0.001; retrospective cohort study: *I*^*2*^ = 96.7%, *P* < 0.001). The pooled results of subgroup analysis were consistent with the overall results, showing that non-survivors presented higher Ang-2 levels than survivors (Supplementary Material 5).

13 studies were conducted in Asia, and 15 studies were conducted in Europe or other continents. Subgroup analysis indicated that the region was not a source of heterogeneity (Non-Asia: *I*^*2*^ = 90.4%, *P* < 0.001; Asia: *I*^*2*^ = 94.7%, *P* < 0.001). Consistent with the overall findings, the pooled results of the subgroup analysis showed higher Ang-2 levels in non-survivors compared to survivors (Supplementary Material 5).

Sensitivity analysis confirmed the robustness of the results (Supplementary Material 6). Funnel plots and Egger’s test (t = 2.28, *P* = 0.031) suggested publication bias. Comparison of the funnel plots before and after the trim-and-fill method revealed no new literature, maintaining the consistency and reliability of the study results (Supplementary Material 6).

### Evaluation of Ang-2 as a risk factor for mortality

**Fig. 3:**
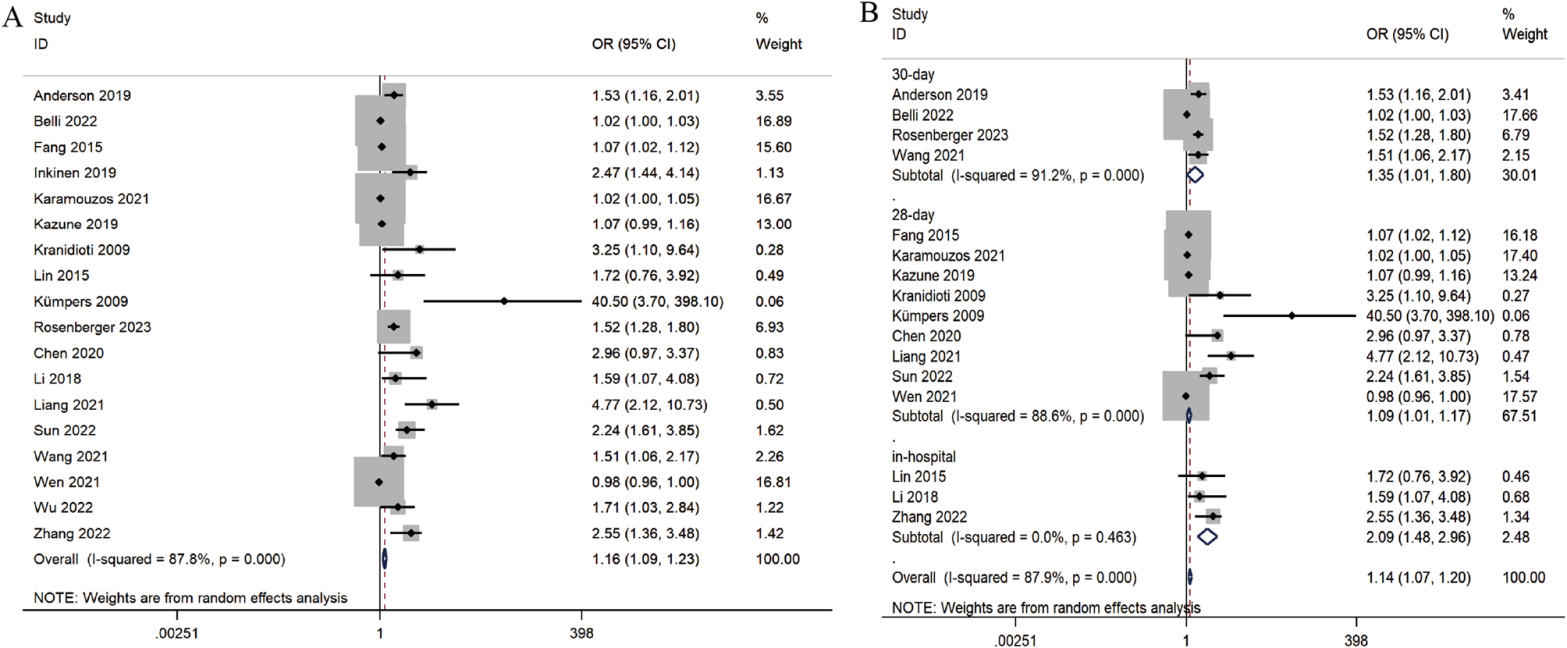
Forest plots of the association between Ang-2 and mortality in septic adult patients. When OR is greater than 1, Ang-2 is a risk factor for poor outcome, otherwise, it is a protective factor. **A** Forest plot of the association between Ang-2 and mortality. **B** Forest plot of the association between Ang-2 and mortality (subgroup analysis according to follow-up duration)

We incorporated 18 studies to evaluate the association between Ang-2 and mortality in septic adult patients. The comprehensive analysis revealed a correlation between these two variables (OR = 1.16, 95% CI 1.09–1.23, *P* < 0.001) (Fig. 3A). These studies were divided into three subgroups based on the follow-up duration (Fig. 3B). Notably, there was considerable heterogeneity among the studies (*I*^*2*^ = 87.8%, *P* < 0.001). According to the sensitivity analysis, three studies [13, 18, 42] had a notable impact on the results (Supplementary Material 6). Nevertheless, the results remained consistent after excluding these three studies, confirming the robustness of the results. Publication bias was analyzed by funnel plots and Egger’s test (t = 8.42, *P* < 0.001), which provided definitive evidence for publication bias. The funnel plot showed significant publication bias (Supplementary Material 6), and the number of missing studies was estimated to be eight by the trim-and-fill method (Supplementary Material 6). The funnel plot was filled to obtain the pooled effect statistic using the random-effects model (OR = 1.11, 95% CI 1.04–1.18, *P* < 0.001).

### Estimation of the predictive performance of Ang-2 for mortality

**Fig. 4:**
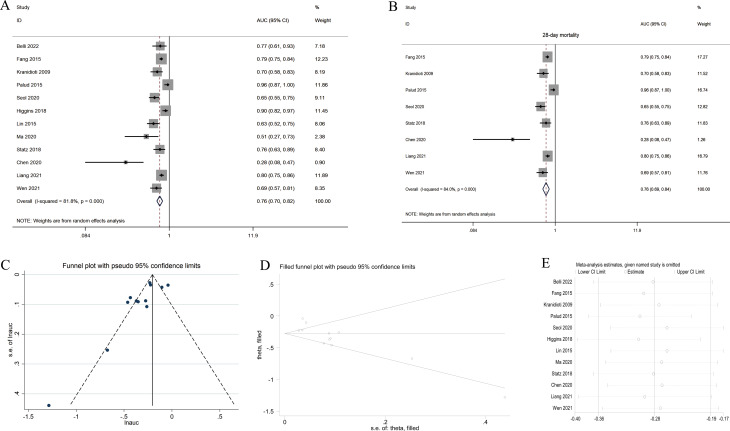
Forest plots of the predictive performance of Ang-2 for mortality in septic adult patients. The closer the value of AUC is to 1, the higher the predictive performance of Ang-2 as a prognostic biomarker. **A** Forest plot of the predictive performance of Ang-2 for mortality. **B** Forest plot of the predictive performance of Ang-2 for mortality (subgroup analysis according to follow-up duration). **C** Funnel plot. **D** Filled funnel plot. **E** Sensitivity analysis

16 of the included studies analyzed the diagnostic power of Ang-2 in predicting mortality, and 12 of these studies could be used for quantitative meta-analysis. Pooled analysis revealed an overall AUC value of 0.76 (95% CI 0.70–0.82, *P* < 0.001) (Fig. 4A). Eight studies evaluated the predictive performance of Ang-2 in predicting 28-day mortality, and the pooled analysis revealed an overall AUC value of 0.76 (95% CI 0.69–0.84, *P* < 0.001) (Fig. 4B). There was considerable heterogeneity between studies [(*I*^*2*^ = 81.8%, *P* < 0.001) (Fig. 4A), (*I*^*2*^ = 84.0%, *P* < 0.001) (Fig. 4B)], necessitating the use of the random-effects model. Furthermore, sensitivity analysis revealed that the results were robust (Fig. 4E). According to the funnel plot and Egger’s test (t = -2.68, *P* = 0.023), the existence of publication bias was confirmed. Comparison of the funnel plots before and after the trim-and-fill method revealed no new literature (Fig. 4C and D), and the recalculated overall effect statistic was 0.76 (95% CI 0.70–0.83, *P* < 0.001).

### Ang/Tie-2 signaling pathway

In this meta-analysis, we included a total of 33 studies that used Ang-2 to predict mortality in septic adult patients, four of which compared Ang-2 with other molecules in the Ang/Tie-2 signaling pathway. The quantity of pertinent studies is insufficient for a comprehensive quantitative meta-analysis, and Table 2 displays the data.

**Table 2:** Comparison of members of Ang/Tie-2 signaling pathway as prognostic biomarkers

	Study	Ang-2	Ang-1	Tie-2	Ang-2/Ang-1	Ang-1/Tie-2
OR (95%CI)	Belli 2022	1.02 (1.00, 1.03)	1.00 (0.99, 1.01)	-	-	-
	Fang 2015	1.07 (1.02, 1.12)	0.94 (0.80, 1.10)	1.00 (1.00, 1.07)	0.99 (0.91, 1.07)	0.01 (0.01, 0.01)
	Lin 2015	1.72 (0.76, 3.92)	2.57 (1.12, 5.90)	-	-	-
AUC (95%CI)	Fang 2015	0.79 (0.75, 0.84)	0.78 (0.73, 0,82)	0.80 (0.76, 0.85)	0.85 (0.81, 0.88)	0.81 (0.76–0.85)
	Seol 2020	0.65 (0.55, 0.75)	-	-	0.74 (0.65, 0.82)	-
	Lin 2015	0.63 (0.52, 0.75)	0.74 (0.73, 0.85)	-	-	-

## Discussion

In this systematic review, we aimed to analyze the association between Ang-2 and mortality in septic adult patients, and ultimately 33 studies were included regardless of the follow-up duration. Our data showed that non-survivors exhibited higher Ang-2 levels compared to survivors. Moreover, Ang-2 was demonstrated to be able to predict mortality in septic adult patients. Therefore, Ang-2 may assist in the early identification of patients at high risk of death from sepsis.

As a member of the angiopoietins family, Ang-2 functions through the Ang/Tie-2 signaling pathway [48]. Many preclinical studies have emphasized the importance of the Ang/Tie-2 pathway in the onset and progression of sepsis [49–51]. A novel antibody, known as the ANG2-binding and TIE2-activating antibody (ABTAA), has been proven to be highly effective in protecting the endothelial barrier. It helps prevent blood components and pathogens from entering surrounding tissues, protects the integrity of endothelial glycocalyx and microvasculature, alleviates vascular leakage, and reduces cytokine storms in sepsis [52]. Ang-2 has been widely studied as a key molecule in the Ang/Tie-2 pathway. Higgins et al. [30] revealed a pivotal contribution of Ang-2 to the process of sepsis, serving as the key molecule linking coagulation, inflammation, complement, and other systems. Elevated Ang-2 levels in sepsis lead to increased vascular endothelial permeability, which may aggravate severity and cause organ dysfunction and mortality [28, 49, 53]. Our meta-analysis demonstrated the prognostic significance of Ang-2 in predicting mortality in septic adult patients. We also compared Ang-2 with other molecules in the Ang/Tie-2 pathway. However, the number of relevant studies on this comparison was insufficient for a comprehensive quantitative synthesis.

To our knowledge, Pregernig et al. [11] reported a meta-analysis evaluating Ang-2 as a biomarker for predicting mortality in adult patients with sepsis. Both our meta-analysis and the one conducted by Pregernig et al. [11] reached the consensus that Ang-2 was correlated with mortality in septic adult patients. Our meta-analysis, however, differs from that of Pregernig et al. in terms of inclusion and exclusion criteria, follow-up duration for prognosis, types of study design, and the languages of the included studies. Of particular importance, we included sepsis patients diagnosed according to sepsis 3.0 criteria. In addition, we attempted to identify the molecule with the best prognosis-predicting performance in the Ang/Tie-2 signaling pathway.

Our study found that Ang-2 was associated with 28-day mortality, 30-day mortality, in-hospital mortality, and 90-day mortality in septic adult patients. Ang-2 predicted 28-day mortality with a reasonable degree of accuracy. We analyzed the differences in Ang-2 levels between survivors and non-survivors in the context of various sepsis diagnostic criteria. The results showed that the Ang-2 levels were higher in non-survivors in the subgroups of sepsis 1.0 and sepsis 3.0 criteria. However, there was no difference in the subgroup of sepsis 2.0 criteria. This may be related to the limited number of clinical studies due to the complexity of sepsis 2.0 criteria [54]. These results should be interpreted with caution because of the high degree of heterogeneity among the included studies. To explore the sources of heterogeneity, we conducted subgroup analysis. There are differences in genetic differences, environmental factors, and disease susceptibility in different regions, which may lead to the high heterogeneity of research results. Through subgroup analysis, we showed that regions were not the source of high heterogeneity. Villar et al. [36] compared the difference of Ang-2 levels in plasma and serum in sepsis patients requiring mechanical ventilation and found that the difference was statistically significant, which provided a reference for us to identify a second subgroup. The results of subgroup analysis indicated that specimen source (plasma or serum) was not a factor contributing to the high heterogeneity. The other two subgroups are follow-up duration and types of study. By subgroup analysis, we did not find the source of heterogeneity, but the sensitivity analysis results showed that our study results were robust and reliable. Furthermore, we sought to identify the molecule with the highest prognostic predictive efficacy in the Ang/Tie-2 signaling pathway. Although the quantities were inadequate for quantitative analysis, our summary results showed that alternative molecules or combinations of molecules did not provide substantial advantages over Ang-2 alone. Ang-2, being the most extensively studied molecule in the Ang/Tie-2 pathway, holds significant promise in predicting the unfavorable outcome of sepsis.

There are several limitations in our study. First, the included studies were all observational due to the nature of the research content. Second, due to publication bias and significant heterogeneity among the included studies, these results should be interpreted with caution. We tried to find the source of heterogeneity through sensitivity analysis and subgroup analysis, but failed. Nonetheless, the filled funnel plot and sensitivity analysis confirmed the robustness and reliability of the results to some degree. Third, we had planned to compare Ang-2 with Ang-1 or Tie-2, or a combination of them, to confirm the hypothesis, but quantitative analysis was not possible due to the lack of relevant data. Finally, some of the data was only published in Chinese.

## Conclusion

Our meta-analysis suggests that Ang-2 is able to predict mortality in septic adult patients. It may facilitate the early identification of sepsis patients at high risk of poor prognosis. Additional investigations are needed to establish the optimal cut-off value prior to widespread clinical application.

## Electronic supplementary material

Below is the link to the electronic supplementary material.

Supplementary Material 1: PRISMA checklist.

Supplementary Material 2: Search Strategy.

Supplementary Material 3: Characteristics of included studies.

Supplementary Material 4: Quality assessment.

Supplementary Material 5: Subgroup analysis.

Supplementary Material 6: Funnel plot and Sensitivity analysis.

## Data Availability

All data generated and/or analyzed during the current study are included within the published article and its additional files.

## Abbreviations

ABTAA: ANG2-binding and TIE2-activating antibody
AKI: Acute kidney injury
Ang-2: Angiopoietin-2
ARDS: Acute respiratory distress syndrome
AUC: Area under the curve
CI: Confidence intervals
CMeSH: Chinese medical subject headings
HR: Hazard ratios
MC: Multiple center
MD: Mean differences
MeSH: Medical subject headings
NOS: Newcastle-Ottawa scale
OR: Odds ratios
PC: Prospective cohort study
PRISMA: Preferred Reporting Items for Systematic Reviews and Meta-Analyses
RC: Retrospective cohort study
SC: Single center
SIRS: Systemic inflammatory response syndrome
SMD: Standardized mean differences
SSC: Surviving Sepsis Campaign

## References

[1] Singer M, Deutschman CS, Seymour CW, Shankar-Hari M, Annane D, Bauer M, et al. The Third International Consensus definitions for Sepsis and septic shock (Sepsis-3). JAMA. 2016;315(8):801–10. 26903338 10.1001/jama.2016.0287 PMC4968574

[2] Evans L, Rhodes A, Alhazzani W, Antonelli M, Coopersmith CM, French C, et al. Surviving sepsis campaign: international guidelines for management of sepsis and septic shock 2021. Intensive Care Med. 2021;47(11):1181–247. 34599691 10.1007/s00134-021-06506-y PMC8486643

[3] Ait-Oufella H, Maury E, Lehoux S, Guidet B, Offenstadt G. The endothelium: physiological functions and role in microcirculatory failure during severe sepsis. Intensive Care Med. 2010;36(8):1286–98. 20443110 10.1007/s00134-010-1893-6

[4] Joffre J, Hellman J, Ince C, Ait-Oufella H. Endothelial responses in Sepsis. Am J Respir Crit Care Med. 2020;202(3):361–70. 32101446 10.1164/rccm.201910-1911TR

[5] Lukasz A, Hillgruber C, Oberleithner H, Kusche-Vihrog K, Pavenstädt H, Rovas A, et al. Endothelial glycocalyx breakdown is mediated by angiopoietin-2. Cardiovasc Res. 2017;113(6):671–80. 28453727 10.1093/cvr/cvx023

[6] Saravi B, Goebel U, Hassenzahl LO, Jung C, David S, Feldheiser A, et al. Capillary leak and endothelial permeability in critically ill patients: a current overview. Intensive Care Med Exp. 2023;11(1):96. 38117435 10.1186/s40635-023-00582-8 PMC10733291

[7] Wollborn J, Zhang Z, Gaa J, Gentner M, Hausmann C, Saenger F, et al. Angiopoietin-2 is associated with capillary leak and predicts complications after cardiac surgery. Ann Intensive Care. 2023;13(1):70. 37552379 10.1186/s13613-023-01165-2 PMC10409979

[8] Wollborn J, Hassenzahl LO, Reker D, Staehle HF, Omlor AM, Baar W, et al. Diagnosing capillary leak in critically ill patients: development of an innovative scoring instrument for non-invasive detection. Ann Intensive Care. 2021;11(1):175. 34910264 10.1186/s13613-021-00965-8 PMC8674404

[9] Thamm K, David S. Role of angiopoietin-2 in infection - A double-edged sword? Cytokine. 2016;83:61–3. 27038015 10.1016/j.cyto.2016.03.019

[10] Scholz A, Plate KH, Reiss Y. Angiopoietin-2: a multifaceted cytokine that functions in both angiogenesis and inflammation. Ann N Y Acad Sci. 2015;1347:45–51. 25773744 10.1111/nyas.12726

[11] Pregernig A, Müller M, Held U, Beck-Schimmer B. Prediction of mortality in adult patients with sepsis using six biomarkers: a systematic review and meta-analysis. Ann Intensive Care. 2019;9(1):125. 31705327 10.1186/s13613-019-0600-1 PMC6841861

[12] Beurskens DM, Bol ME, Delhaas T, van de Poll MC, Reutelingsperger CP, Nicolaes GA, et al. Decreased endothelial glycocalyx thickness is an early predictor of mortality in sepsis. Anaesth Intensive Care. 2020;48(3):221–8. 32486831 10.1177/0310057X20916471 PMC7328096

[13] Belli OE, Campolo J, Vallerio P, Musca F, Moreo A, Maloberti A, et al. Biochemical but not imaging parameters are predictive of outcome in septic shock: a pilot study. Cardiovasc Ultrasound. 2022;20(1):6. 35331262 10.1186/s12947-022-00276-3 PMC8943962

[14] Karamouzos V, Giamarellos-Bourboulis EJ, Velissaris D, Gkavogianni T, Gogos C. Cytokine production and outcome in MDR versus non-MDR gram-negative bacteraemia and sepsis. Infect Dis (Lond). 2021;53(10):764–71. 34137348 10.1080/23744235.2021.1925738

[15] Page MJ, McKenzie JE, Bossuyt PM, Boutron I, Hoffmann TC, Mulrow CD, et al. The PRISMA 2020 statement: an updated guideline for reporting systematic reviews. BMJ. 2021;372:n71. 33782057 10.1136/bmj.n71 PMC8005924

[16] McGrath S, Zhao X, Steele R, Thombs BD, Benedetti A. Estimating the sample mean and standard deviation from commonly reported quantiles in meta-analysis. Stat Methods Med Res. 2020;29(9):2520–37. 32292115 10.1177/0962280219889080 PMC7390706

[17] Higgins J. Cochrane handbook for systematic reviews of interventions. Cochrane Collaboration and John Wiley & Sons Ltd.; 2008.

[18] Anderson BJ, Calfee CS, Liu KD, Reilly JP, Kangelaris KN, Shashaty MGS, et al. Plasma sTNFR1 and IL8 for prognostic enrichment in sepsis trials: a prospective cohort study. Crit Care. 2019;23(1):400. 31818332 10.1186/s13054-019-2684-2 PMC6902425

[19] Davis JS, Yeo TW, Piera KA, Woodberry T, Celermajer DS, Stephens DP, et al. Angiopoietin-2 is increased in sepsis and inversely associated with nitric oxide-dependent microvascular reactivity. Crit Care. 2010;14(3):R89. 20482750 10.1186/cc9020 PMC2911723

[20] Fang Y, Li C, Shao R, Yu H, Zhang Q, Zhao L. Prognostic significance of the angiopoietin-2/angiopoietin-1 and angiopoietin-1/Tie-2 ratios for early sepsis in an emergency department. Crit Care. 2015;19:367. 26463042 10.1186/s13054-015-1075-6 PMC4604731

[21] Inkinen N, Pettilä V, Lakkisto P, Kuitunen A, Jukarainen S, Bendel S, et al. Association of endothelial and glycocalyx injury biomarkers with fluid administration, development of acute kidney injury, and 90-day mortality: data from the FINNAKI observational study. Ann Intensive Care. 2019;9(1):103. 31512003 10.1186/s13613-019-0575-y PMC6738365

[22] Kazune S, Caica A, Volceka K, Suba O, Rubins U, Grabovskis A. Relationship of mottling score, skin microcirculatory perfusion indices and biomarkers of endothelial dysfunction in patients with septic shock: an observational study. Crit Care. 2019;23(1):311. 31511042 10.1186/s13054-019-2589-0 PMC6739999

[23] Kranidioti H, Orfanos SE, Vaki I, Kotanidou A, Raftogiannis M, Dimopoulou I, et al. Angiopoietin-2 is increased in septic shock: evidence for the existence of a circulating factor stimulating its release from human monocytes. Immunol Lett. 2009;125(1):65–71. 19539650 10.1016/j.imlet.2009.06.006

[24] Palud A, Parmentier-Decrucq E, Pastre J, De Freitas Caires N, Lassalle P, Mathieu D. Evaluation of endothelial biomarkers as predictors of organ failures in septic shock patients. Cytokine. 2015;73(2):213–8. 25794660 10.1016/j.cyto.2015.02.013

[25] Ricciuto DR, dos Santos CC, Hawkes M, Toltl LJ, Conroy AL, Rajwans N, et al. Angiopoietin-1 and angiopoietin-2 as clinically informative prognostic biomarkers of morbidity and mortality in severe sepsis. Crit Care Med. 2011;39(4):702–10. 21242795 10.1097/CCM.0b013e318206d285

[26] Seol CH, Yong SH, Shin JH, Lee SH, Leem AY, Park MS, et al. The ratio of plasma angiopoietin-2 to angiopoietin-1 as a prognostic biomarker in patients with sepsis. Cytokine. 2020;129:155029. 32059166 10.1016/j.cyto.2020.155029

[27] Sexton T, Chalhoub G, Ye S, Morris W, Annabathula R, Dugan A, et al. Autotaxin Activity predicts 30-Day mortality in Sepsis patients and correlates with platelet count and vascular dysfunction. Shock. 2020;54(6):738–43. 32826822 10.1097/SHK.0000000000001569

[28] Siner JM, Bhandari V, Engle KM, Elias JA, Siegel MD. Elevated serum angiopoietin 2 levels are associated with increased mortality in sepsis. Shock. 2009;31(4):348–53. 18791490 10.1097/SHK.0b013e318188bd06

[29] Walborn A, Hoppensteadt D, Rondina MT, Fareed J. Development of an algorithm to predict mortality in patients with sepsis and coagulopathy. Blood. 2018;132. 10.1177/1076029620902849 PMC7288806 32129085

[30] Higgins SJ, De Ceunynck K, Kellum JA, Chen X, Gu X, Chaudhry SA, et al. Tie2 protects the vasculature against thrombus formation in systemic inflammation. J Clin Invest. 2018;128(4):1471–84. 29360642 10.1172/JCI97488 PMC5873892

[31] Lin SM, Chung FT, Kuo CH, Chou PC, Wang TY, Chang PJ, et al. Circulating angiopopietin-1 correlates with the clinical course of multiple organ dysfunction syndrome and mortality in patients with severe sepsis. Med (Baltim). 2015;94(20):e878. 10.1097/MD.0000000000000878 PMC4602874 25997069

[32] Kümpers P, van Meurs M, David S, Molema G, Bijzet J, Lukasz A, et al. Time course of angiopoietin-2 release during experimental human endotoxemia and sepsis. Crit Care. 2009;13(3):R64. 19416526 10.1186/cc7866 PMC2717419

[33] Ma S, Zhao ML, Wang K, Yue YF, Sun RQ, Zhang RM, et al. Association of Ang-2, vWF, and EVLWI with risk of mortality in sepsis patients with concomitant ARDS: a retrospective study. J Formos Med Assoc. 2020;119(5):950–6. 31822372 10.1016/j.jfma.2019.11.005

[34] Rosenberger CM, Wick KD, Zhuo H, Wu N, Chen Y, Kapadia SB, et al. Early plasma angiopoietin-2 is prognostic for ARDS and mortality among critically ill patients with sepsis. Crit Care. 2023;27(1):234. 37312169 10.1186/s13054-023-04525-3 PMC10261831

[35] Statz S, Sabal G, Walborn A, Williams M, Hoppensteadt D, Mosier M, et al. Angiopoietin 2 levels in the risk stratification and mortality outcome prediction of Sepsis-Associated Coagulopathy. Clin Appl Thromb Hemost. 2018;24(8):1223–33. 29996658 10.1177/1076029618786029 PMC6714761

[36] Villar J, Herrán-Monge R, González-Higueras E, Prieto-González M, Ambrós A, Rodríguez-Pérez A, et al. Clinical and biological markers for predicting ARDS and outcome in septic patients. Sci Rep. 2021;11(1):22702. 34811434 10.1038/s41598-021-02100-w PMC8608812

[37] Parikh SM, Mammoto T, Schultz A, Yuan HT, Christiani D, Karumanchi SA, et al. Excess circulating angiopoietin-2 may contribute to pulmonary vascular leak in sepsis in humans. PLoS Med. 2006;3(3):e46. 16417407 10.1371/journal.pmed.0030046 PMC1334221

[38] Chen YQ, Huang X, Kong GQ, Liu XL, Hao D. Significance of high mobility group box 1, Von Willebrand factor and other cytokines in the evaluation of severity and prognosis of sepsis patients. Zhonghua Wei Zhong Bing Ji Jiu Yi Xue. 2020;32(8):933–7. 32912405 10.3760/cma.j.cn121430-20200428-00346

[39] Guan YD, Tang HF, Liu CM, Wang YJ, Zhao W, Li JJ, et al. Correlation of serum VEGFR-2,ANG-2,VE-Cad levels with severity and prognosis in patients with septic shock. Lab Med Clin. 2021;18(18):2708–11.

[40] Lei XH, Li T. Serum Angiopoietin-2,Citrulline and procalcitonin for diagnosis and prognosis evaluation in patients with Sepsis and Acute Respiratory Distress Syndrome. J Nanchang University: Med Sci. 2022;62(1):43–7.

[41] li CS, Chang C, Dai HL, Guo H, Xiao M. Value of EVLWI and Ang-2 in predicting prognosis of sepsis complicated with ARDS. J Crit Care Intern Med. 2018;24(5):372–4.

[42] Liang XL. Prognostic value of serum Ang2 combined with SOFA score in patients with septic shock. Mod Practical Med. 2021;33(9):1175–6.

[43] Sun HZ, Sun HY, Li YP. Relationship between serum levels of sTM,suPAR and Ang-2 and inflammatory factors and prognosis in patients with sepsis complicated with ARDS. Lab Med Clin. 2022;19(8):1075–9.

[44] Wang NJ, Wu HF, Zhang JY. Analysis of correlation between sTREM-1,Ang-2 and PCT and prognosis of patients with sepsis. J Trop Med. 2021;21(12):1568–71.

[45] Wen J, Zhang YJ, Chen Q, Tan YP. Changes of serum VE-Cad and Ang-2 levels in patients with sepsis complicated by ARDS and their relationship with prognosis. J Hebei Med Univ. 2021;42(4):376–985.

[46] Wu LJ. Correlation analysis of changes of sTREM-1,Ang-2 and PCT levels before and after treatment and prognosis in sepsis patients. Med Lab Sci Clin. 2022;33(12):32–5.

[47] Zhang Y, Wang J. Expression of serum vascular endothelial cadherin and angiopoietin 2 in sepsis patients complicated with acute respiratory distress syndrome and their correlation with prognosis. Shaanxi Med J. 2022;51(2):176–990.

[48] Siner JM. A tale of two ligands: angiopoietins, the endothelium, and outcomes. Crit Care. 2013;17(5):1007. 24131798 10.1186/cc13066 PMC4057381

[49] David S, Mukherjee A, Ghosh CC, Yano M, Khankin EV, Wenger JB, et al. Angiopoietin-2 may contribute to multiple organ dysfunction and death in sepsis*. Crit Care Med. 2012;40(11):3034–41. 22890252 10.1097/CCM.0b013e31825fdc31 PMC3705559

[50] Ziegler T, Horstkotte J, Schwab C, Pfetsch V, Weinmann K, Dietzel S, et al. Angiopoietin 2 mediates microvascular and hemodynamic alterations in sepsis. J Clin Invest. 2013;123(8):3436–45. 23863629 10.1172/JCI66549 PMC3726157

[51] Stiehl T, Thamm K, Kaufmann J, Schaeper U, Kirsch T, Haller H, et al. Lung-targeted RNA interference against angiopoietin-2 ameliorates multiple organ dysfunction and death in sepsis. Crit Care Med. 2014;42(10):e654–62. 25083983 10.1097/CCM.0000000000000524

[52] Han S, Lee SJ, Kim KE, Lee HS, Oh N, Park I, et al. Amelioration of sepsis by TIE2 activation-induced vascular protection. Sci Transl Med. 2016;8(335):335ra55. 27099174 10.1126/scitranslmed.aad9260

[53] Lymperopoulou K, Velissaris D, Kotsaki A, Antypa E, Georgiadou S, Tsaganos T, et al. Angiopoietin-2 associations with the underlying infection and sepsis severity. Cytokine. 2015;73(1):163–8. 25748839 10.1016/j.cyto.2015.01.022

[54] Levy MM, Fink MP, Marshall JC, Abraham E, Angus D, Cook D et al. 2001 SCCM/ESICM/ACCP/ATS/SIS International Sepsis Definitions Conference. Crit Care Med. 2003;31(4):1250-6. 10.1097/01.CCM.0000050454.01978.3B 12682500

