# Supplementary material for: Angiopoietin-2 as a prognostic biomarker in septic adult patients: a systemic review and meta-analysis
Source: Ann Intensive Care. 2024 Nov 10;14:169. doi: 10.1186/s13613-024-01393-0 (PMC11551087; doi:10.1186/s13613-024-01393-0)
Supplement: Supplementary file 2 — Supplementary Material 2: Search Strategy. [file 13613_2024_1393_MOESM2_ESM.docx]

**PubMed**

Searched June 21^th^, 2022 Records identified: 100

Updated search July 16^th^, 2023 Records identified: 112

((((sepsis[MeSH Terms]) OR (((((((((((((((((Bloodstream Infection[Title/Abstract]) OR (Bloodstream Infections[Title/Abstract])) OR (Infection, Bloodstream[Title/Abstract])) OR (Pyemia[Title/Abstract])) OR (Pyemias[Title/Abstract])) OR (Pyohemia[Title/Abstract])) OR (Pyohemias[Title/Abstract])) OR (Pyaemia[Title/Abstract])) OR (Pyaemias[Title/Abstract])) OR (Septicemia[Title/Abstract])) OR (Septicemias[Title/Abstract])) OR (Poisoning, Blood[Title/Abstract])) OR (Blood Poisoning[Title/Abstract])) OR (Blood Poisonings[Title/Abstract])) OR (Poisonings, Blood[Title/Abstract])) OR (Severe Sepsis[Title/Abstract])) OR (Sepsis, Severe[Title/Abstract]))) OR ("Shock, Septic"[Mesh])) OR (((((((((((Septic Shock[Title/Abstract]) OR (Shock, Toxic[Title/Abstract])) OR (Toxic Shock Syndrome[Title/Abstract])) OR (Shock Syndrome, Toxic[Title/Abstract])) OR (Toxic Shock Syndromes[Title/Abstract])) OR (Toxic Shock[Title/Abstract])) OR (Shock, Endotoxic[Title/Abstract])) OR (Endotoxin Shock[Title/Abstract])) OR (Endotoxin Shocks[Title/Abstract])) OR (Shock, Endotoxin[Title/Abstract])) OR (Shocks, Endotoxin[Title/Abstract]))) AND (("Angiopoietin-2"[Mesh]) OR (Angiopoietin 2[Title/Abstract]))) AND (("Prognosis"[Mesh]) OR (((((prognos*[Title/Abstract]) OR (predict*[Title/Abstract])) OR (outcome*[Title/Abstract])) OR (mortality[Title/Abstract])) OR (surviv*[Title/Abstract])))

**Embase**

Searched June 21th, 2022 Records identified: 250

Updated search July 16th, 2023 Records identified: 267

#1: ‘sepsis’/exp

#2: ‘bloodstream Infection’: ab, ti

#3: ‘infection, bloodstream’: ab, ti

#4: ‘pyemia’: ab, ti

#5: ‘pyemias’: ab, ti

#6: ‘pyohemias’: ab, ti

#7: ‘pyaemia’: ab, ti

#8: ‘septicemia’: ab, ti

#9: ‘septicemias’: ab, ti

#10: ‘poisoning, blood’: ab, ti

#11: ‘blood poisoning’: ab, ti

#12: ‘blood poisonings’: ab, ti

#13: ‘poisonings, blood’: ab, ti

#14: ‘severe sepsis’: ab, ti

#15: ‘sepsis, severe’: ab, ti

#16: ‘septic shock’/exp

#17: ‘septic shock’: ab, ti

#18: ‘toxic shock syndrome’: ab, ti

#19: ‘shock syndrome, toxic’: ab, ti
#20: ‘toxic shock syndromes’: ab, ti
#21: ‘shock, endotoxic’: ab, ti

#22: ‘endotoxic shock’: ab, ti

#23: ‘endotoxin shocks’: ab, ti

#24: ‘shocks, endotoxin’: ab, ti

#25: #1 OR #2 OR #3 OR #4 OR #5 OR #6 OR #7 OR #8 OR #9 OR #10 OR #11 OR #12 OR #13 OR #14 OR #15 OR #16 OR #17 OR #18 OR #19 OR #20 OR #21 OR #22 OR #23 OR #24

#26: ‘angiopoietin 2’/exp

#27: ‘angiopoietin 2’: ab, ti

#28: #26 OR #27

#29: ‘prognosis’/exp

#30: ‘prognosis’: ab, ti

#31: ‘prognostic assessment’: ab, ti

#32: ‘prognostic value’: ab, ti

#33: ‘prognostic index’: ab, ti

#34: ‘prognostic factor’: ab, ti

#35: ‘prognostic model’: ab, ti

#36: ‘prediction’: ab, ti

#37: ‘prediction and forecasting’: ab, ti

#38: ‘predictive value’: ab, ti

#39: ‘predictor variable’: ab, ti

#40: ‘predictive validity’: ab, ti

#41: ‘predictive model’: ab, ti

#42: ‘outcome’: ab, ti

#43: ‘outcome assessment’: ab, ti

#44: ‘outcomes research’: ab, ti

#45: ‘outcome variable’: ab, ti

#46: ‘mortality’: ab, ti

#47: ‘cause of death’: ab, ti

#48: ‘mortality rate’: ab, ti

#49: ‘mortality risk’: ab, ti

#50: ‘survival’: ab, ti

#51: ‘survival rate’: ab, ti

#52: ‘survival time’: ab, ti

#53: ‘survivor’: ab, ti

#54: #29 OR #30 OR #31 OR #32 OR #33 OR #34 OR #35 OR #36 OR #37 OR #38 OR #39 OR #40 OR #41 OR #42 OR #43 OR #44 OR #45 OR #46 OR #47 OR #48 OR #49 OR #50 OR #51 OR #52 OR #53

#55: #25 AND #28 AND #54

**Cochranelibrary**

Searched June 21th, 2022 Records identified: 17

Updated search July 16th, 2023 Records identified: 23

#1: MeSH descriptor: [sepsis] explode all trees

#2: (Bloodstream Infection): ti, ab, kw OR (Bloodstream Infections): ti, ab, kw OR (Infection, Bloodstream): ti, ab, kw OR (Pyemia): ti, ab, kw OR (Pyemias): ti, ab, kw

#3: (Pyohemia): ti, ab, kw OR (Pyohemias): ti, ab, kw OR (Pyaemia): ti, ab, kw OR (Pyaemias): ti, ab, kw OR (Septicemia): ti, ab, kw

#4: (Septicemias): ti, ab, kw OR (Poisoning, Blood): ti, ab, kw OR (Blood Poisoning): ti, ab, kw OR (Blood Poisonings): ti, ab, kw OR (Poisonings, Blood): ti, ab, kw

#5: (Severe Sepsis): ti, ab, kw OR (Sepsis, Severe): ti, ab, kw

#6: MeSH descriptor: [Shock, Septic] explode all trees

#7: (Septic Shock): ti, ab, kw OR (Shock, Toxic): ti, ab, kw OR (Toxic Shock Syndrome): ti, ab, kw OR (Shock Syndrome, Toxic): ti, ab, kw OR (Toxic Shock Syndromes): ti, ab, kw

#8: (Toxic Shock): ti, ab, kw OR (Shock, Endotoxic): ti, ab, kw OR (Endotoxin Shock): ti, ab, kw OR (Endotoxin Shocks): ti, ab, kw OR (Shock, Endotoxin): ti, ab, kw

#9: (Shocks, Endotoxin): ti, ab, kw

#10: #1 OR #2 OR #3 OR #4 OR #5 OR #6 OR #7 OR #8 OR #9

#11: MeSH descriptor: [Angiopoietin-2] explode all trees

#12: (“angiopoietin 2”): ti, ab, kw

#13: #11 OR #12

#14: MeSH descriptor: [Prognosis] explode all trees

#15: (prognos*): ti, ab, kw OR (predict*): ti, ab, kw OR (outcome*): ti, ab, kw OR (mortality): ti, ab, kw OR (surviv*): ti, ab, kw

#16: #14 OR #15

#17: #10 AND #13 AND #16

**Similar search strategy is applied to the following 4 Chinese databases:**

**CNKI**

Updated search July 16th, 2023 Records identified: 44

**WanFang**

Updated search July 16th, 2023 Records identified: 14

**CQVIP**

Updated search July 16th, 2023 Records identified: 24

**CBM**

Updated search July 16th, 2023 Records identified: 23
