# Supplementary material for: Angiopoietin-2 as a prognostic biomarker in septic adult patients: a systemic review and meta-analysis
Source: Ann Intensive Care. 2024 Nov 10;14:169. doi: 10.1186/s13613-024-01393-0 (PMC11551087; doi:10.1186/s13613-024-01393-0)
Supplement: Supplementary file 3 — Supplementary Material 3: Characteristics of included studies. [file 13613_2024_1393_MOESM3_ESM.docx]

**Characteristics of included studies**

| **Author** | **Setting**  **(ICU/**  **Ward/ED)** | **Treatment** | **Cut-off value** | **Follow-up** | **Ang-2 assay** | **Sampling time** | **Infection source(%)** | **Host immune status^*^** |
| --- | --- | --- | --- | --- | --- | --- | --- | --- |
| Anderson [18] | ED、ICU | NM | 9761 pg/ml | 30-day | ELISA | within 24h | Lung (45.5) | No |
| Belli [13] | ICU | NM | 33418 pg/ml | 30-day | ELISA | within 24h | Gastrointestinal (29), autoimmune (3), respiratory (20), vascular surgery/limb ischaemia (3), genitourinary (14), orthopaedic/skeletal (3) and haematological (29) | No |
| Beurskens [12] | ICU | NM | NM | in-hospital | ELISA | within 24h | Abdominal (42.9), respiratory (28.6), urinary (9.5), other (19.0) | No |
| Davis [19] | ICU、 Ward | NM | NM | 28-day | ELISA | baseline,  day 2-4 | NM | No |
| Fang [20] | ED | NM | NM | 28-day | ELISA | admission | Lung (52.5), urinary tract (3.9), cerebral (5.9), IAI (12.0), skin and soft tissue (1.4) | yes |
| Inkinen [21] | ICU | Sepsis Guidelines | NM | 90-day | NM | admission | Lungs (47.8), gastrointestinal tract (25.2), urinary tract (7.4), skin and soft tissue (8.9), other (3.1) | No |
| Karamouzos [14] | ICU | NM | NM | 28-day | ELISA | admission | Pyelonephritis (64.1), lung (11.7), bacteraemia (17.2), IAI (6.3), CNS infection (0.8) | yes |
| Kazune [22] | ICU | local protocols | NM | 28-day | ELISA | within 24h | Abdominal (35), urinary (29), respiratory (26), others（10） | No |
| Kranidioti [23] | ICU | NM | 9700 pg/ml | 28-day | ELISA | within 24h | Lung (100) | yes |
| Palud [24] | ICU | NM | 26.78 ng/ml | 28-day | ELISA | admission | Lung (55), skin (15), ENT (15), Others (15) | yes |
| Ricciuto [25] | ICU | NM | NM | 28-day | ELISA | admission | Lung (59), abdomen (16), genitourinary tract (7), skin and soft tissue (7), blood (6),unknown/other (13) | No |
| Seol [26] | ICU | NM | NM | 28-day | Non-  ELISA | within 24h | NM | No |
| Sexton [27] | ICU | NM | NM | 30-day | ELISA | inclusion | Respiratory tract (20.9), urinary tract (19.8), endocarditis (11.6), abscess (8.1), others | NM |
| Siner [28] | ICU | NM | NM | In-hospital | ELISA | Inclusion  (within 48h) | Lung (46), urinary tract (17), skin and soft tissue (8.7), others (20) (endovascular, gastrointestinal, biliary, blood, central nervous system), unknown（8.7） | No |
| Walborn [29] | ICU | NM | NM | 28-day | ELISA | admission | NM | No |
| Higgins [30] | ED | NM | NM | In-hospital | ELISA | admission | NM | NM |
| Lin [31] | ICU | Sepsis Guidelines | NM | in-hospital | ELISA | day1，3，7 | Pneumonia (65.6), primary bloodstream infection（13.5), skin and subcutaneous infection (6.3), urosepsis（11.5), other（3.1） | No |
| Kümpers [32] | ICU | NM | 5.9 ng/ml | 28-day | ELISA | admission,24h,72h | Pneumonia (57.1), peritonitis (19.0), urinary tract infection (9.5), systemic mycosis (9.5), endocarditis (4.8), mediastinitis (4.8) | NM |
| Ma [33] | NM | NM | 2.71 | NM | ELISA | admission,24h,48h | Pulmonary (43.9), abdominal (39.0), other(17.1) | Only malignant tumors were excluded |
| Rosenberger [34] | ED、ICU | NM | NM | 30-day | ELISA | Within 48h | Pulmonary (48), non-pulmonary (40), both pulmonary and non-pulmonary (8), unclear(3) | NM |
| Statz [35] | ICU | NM | NM | 28-day | ELISA | admission | NM | NM |
| Villar [36] | ICU | NM | 4278 pg/ml | 28-day | ELISA | within 24h | Extrapulmonary (60.8), pulmonary (37.1), unknown (2.2) | No |
| Parikh [37] | ICU | NM | NM | in-hospital | ELISA | within 24h | Lung (27), GU (23), catheter (14), abdominal (18), other (18) | No |
| Chen [38] | ICU | NM | 607.6 ng/l | 28-day | ELISA | within 24h | Abdominal (44.4), lung (16.7), hepatobiliary system (19.4), other (19.4) | yes |
| Guan [39] | NM | sepsis consensus | NM | 28-day | ELISA | NM | Blood (34.5), abdominal (31.8), urinary tract (30.0), other (3.6) | Yes |
| Lei [40] | NM | NM | 6.04 mg/l | 28-day | ELISA | within 24h | NM | Yes |
| Li [41] | NM | Sepsis Guidelines | 291.85 ng/l | in-hospital | ELISA | NM | Lung (47.8), abdominal (31.3), other (20.9) | Only malignant tumors were excluded |
| Liang [42] | NM | NM | NM | 28-day | ELISA | within 24h | NM | Yes |
| Sun [43] | NM | Sepsis Guidelines | NM | 28-day | ELISA | Admission | Urinary tract (51.5), CRBSI (67.0), abdominal (35.0), other (12.6) | Only immune system diseases were excluded |
| Wang [44] | NM | Sepsis Guidelines | 11.69 ug/L | 30-day | ELISA | NM | Abdominal (45), lung (31.1), bloodstream (23.9) | Yes |
| Wen [45] | NM | NM | NM | 28-day | ELISA | Within 48h | NM | Only malignant tumors were excluded |
| Wu [46] | NM | Sepsis Guidelines | NM | NM | ELISA | NM | Pneumonia (29.3), peritonitis (37.3), urinary tract (10.7), meningitis (6.7), other (16) | Yes |
| Zhang [47] | NM | Sepsis Guidelines | NM | in-hospital | ELISA | NM | Lung (56.5), abdominal (16.5), urinary tract (14.1), bloodstream (9.4), other (3.5) | No |

*NM* not mention, *IAI* intraabdominal infection, *CNS* central nervous system, *ENT* enteral, *GU* genitourinary, CRBSI catheter-related bloodstream infection.

^*^Whether subjects with immunodeficiency or a history of radiotherapy and chemotherapy for cancer were excluded.
