# Supplementary material for: Angiopoietin-2 as a prognostic biomarker in septic adult patients: a systemic review and meta-analysis
Source: Ann Intensive Care. 2024 Nov 10;14:169. doi: 10.1186/s13613-024-01393-0 (PMC11551087; doi:10.1186/s13613-024-01393-0)
Supplement: Supplementary file 4 — Supplementary Material 4: Quality assessment. [file 13613_2024_1393_MOESM4_ESM.docx]

**Quality scores according to the Newcastle-Ottawa scale**

| **Study** | **Selection** | | | | **Comparability of Cohorts on the Basis of the Design or Analysis** | **OUTCOME** | | | **Scores** |
| --- | --- | --- | --- | --- | --- | --- | --- | --- | --- |
|  | Representativeness of the Exposed Cohort | Selection of the Non-Exposed Cohort | Ascertainment of Exposure | Demonstration That Outcome of Interest Was Not Present at Start of Study |  | Assessment of Outcome | Was Follow-Up Long Enough for Outcomes to Occur | Adequacy of Follow Up of Cohorts |  |
| **Anderson 2019** | ★ | ★ | ★ | ★ | ★ | ★ | ★ | ★ | 8 |
| **Belli 2022** | ★ | ★ | ★ | ★ | ★★ | ★ | ★ | ★ | 9 |
| **Beurskens 2020** | ★ | ★ | ★ | ★ | ★ | ★ | ★ | ★ | 8 |
| **Davis 2010** | ★ | ★ | ★ | ★ |  | ★ | ★ | ★ | 7 |
| **Fang 2015** | ★ | ★ | ★ | ★ |  | ★ | ★ | ★ | 7 |
| **Inkinen 2019** | ★ | ★ | ★ | ★ | ★★ | ★ | ★ | ★ | 9 |
| **Karamouzos 2021** | ★ | ★ | ★ | ★ | ★★ | ★ | ★ | ★ | 9 |
| **Kazune 2019** | ★ | ★ | ★ | ★ | ★ | ★ | ★ | ★ | 8 |
| **Kranidioti 2009** | ★ | ★ | ★ | ★ |  | ★ | ★ | ★ | 7 |
| **Palud 2015** | ★ | ★ | ★ | ★ |  | ★ | ★ | ★ | 7 |
| **Ricciuto 2011** | ★ | ★ | ★ | ★ | ★ | ★ | ★ | ★ | 8 |
| **Seol 2020** | ★ | ★ | ★ | ★ | ★ | ★ | ★ | ★ | 8 |
| **Sexton 2020** | ★ | ★ | ★ | ★ | ★ | ★ | ★ | ★ | 8 |
| **Siner 2009** | ★ | ★ | ★ | ★ |  | ★ | ★ | ★ | 7 |
| **Walborn 2020** | ★ | ★ | ★ | ★ |  | ★ | ★ | ★ | 7 |
| **Higgins 2018** | ★ | ★ | ★ | ★ |  | ★ | ★ | ★ | 7 |
| **Lin 2015** | ★ | ★ | ★ | ★ | ★ | ★ | ★ | ★ | 8 |
| **Kümpers 2009** |  | ★ | ★ | ★ |  | ★ | ★ | ★ | 6 |
| **Ma 2020** | ★ | ★ | ★ | ★ | ★ | ★ |  | ★ | 7 |
| **Rosenberger 2023** | ★ | ★ | ★ | ★ | ★ | ★ | ★ | ★ | 8 |
| **Statz 2018** | ★ | ★ | ★ | ★ | ★ | ★ | ★ | ★ | 8 |
| **Villar 2021** | ★ | ★ | ★ | ★ |  | ★ | ★ | ★ | 7 |
| **Parikh 2006** | ★ | ★ | ★ | ★ |  | ★ | ★ | ★ | 7 |
| **Chen 2020** | ★ | ★ | ★ | ★ |  | ★ | ★ | ★ | 7 |
| **Guan 2021** | ★ | ★ | ★ | ★ |  | ★ | ★ | ★ | 7 |
| **Lei 2022** | ★ | ★ | ★ | ★ |  | ★ | ★ | ★ | 7 |
| **Li 2018** | ★ | ★ | ★ | ★ | ★★ | ★ | ★ | ★ | 9 |
| **Liang 2021** |  | ★ | ★ | ★ | ★ | ★ | ★ | ★ | 7 |
| **Sun 2022** | ★ | ★ | ★ | ★ | ★★ | ★ | ★ | ★ | 9 |
| **Wang 2021** | ★ | ★ | ★ | ★ | ★ | ★ | ★ | ★ | 8 |
| **Wen 2021** | ★ | ★ | ★ | ★ | ★ | ★ | ★ | ★ | 8 |
| **Wu 2022** | ★ | ★ | ★ | ★ | ★ | ★ |  | ★ | 7 |
| **Zhang 2022** | ★ | ★ | ★ | ★ | ★ | ★ | ★ | ★ | 8 |
