# Supplementary material for: Angiopoietin-2 as a prognostic biomarker in septic adult patients: a systemic review and meta-analysis
Source: Ann Intensive Care. 2024 Nov 10;14:169. doi: 10.1186/s13613-024-01393-0 (PMC11551087; doi:10.1186/s13613-024-01393-0)
Supplement: Supplementary file 5 — Supplementary Material 5: Subgroup analysis. [file 13613_2024_1393_MOESM5_ESM.docx]

Forest plot of the levels of angiopoietin-2 between non-survivors and survivors in septic adult patients

- **Fig. 1** subgroup analysis by sample source
- **Fig. 2** subgroup analysis by study type
- **Fig. 3** subgroup analysis by region

**
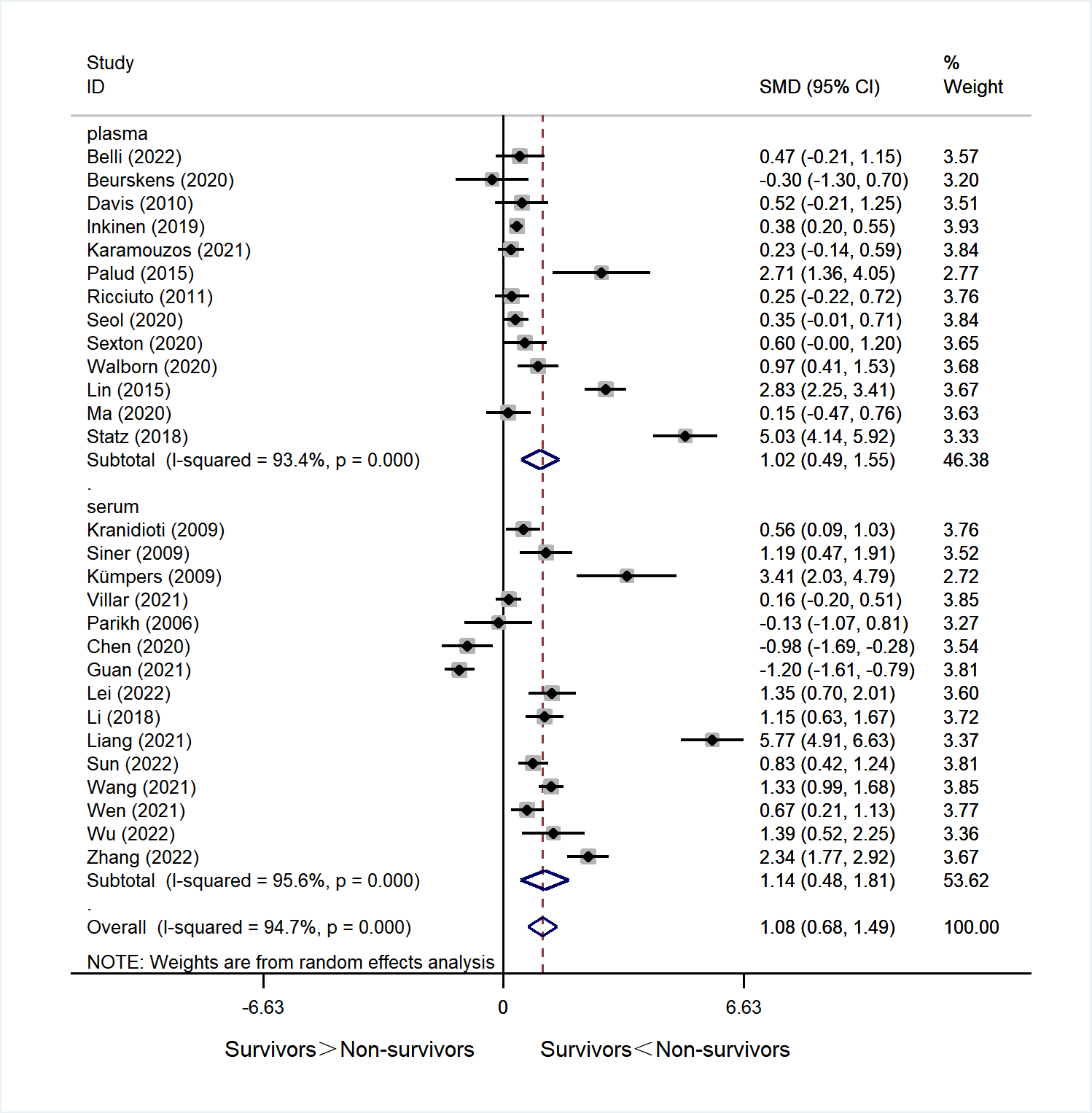
**

**Fig. 1** Angiopoietin-2 levels between non-survivors and survivors in septic adult patients (subgroup analysis by sample source)


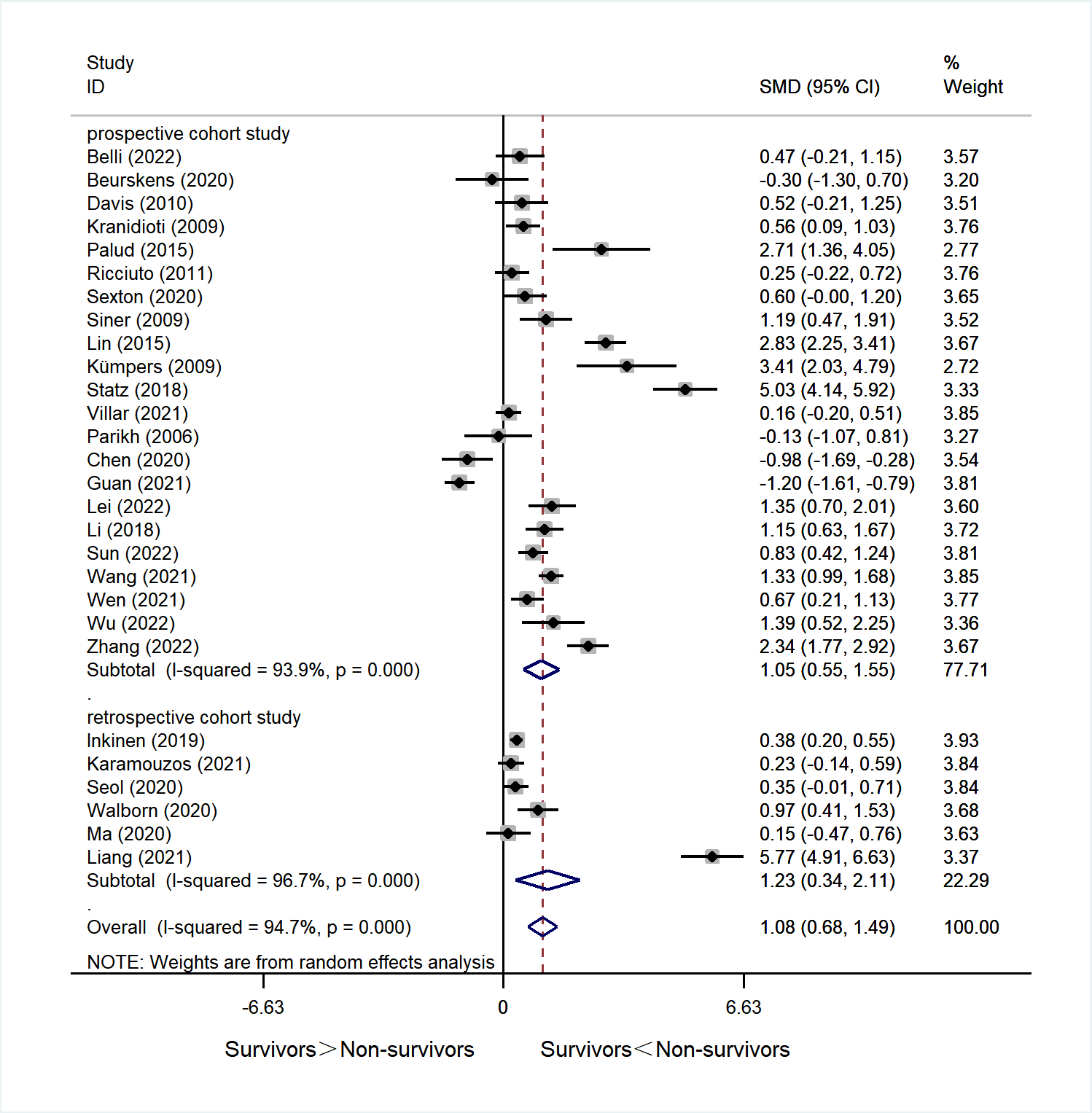
**Fig. 2** Angiopoietin-2 levels between non-survivors and survivors in septic adult patients (subgroup analysis by study type)


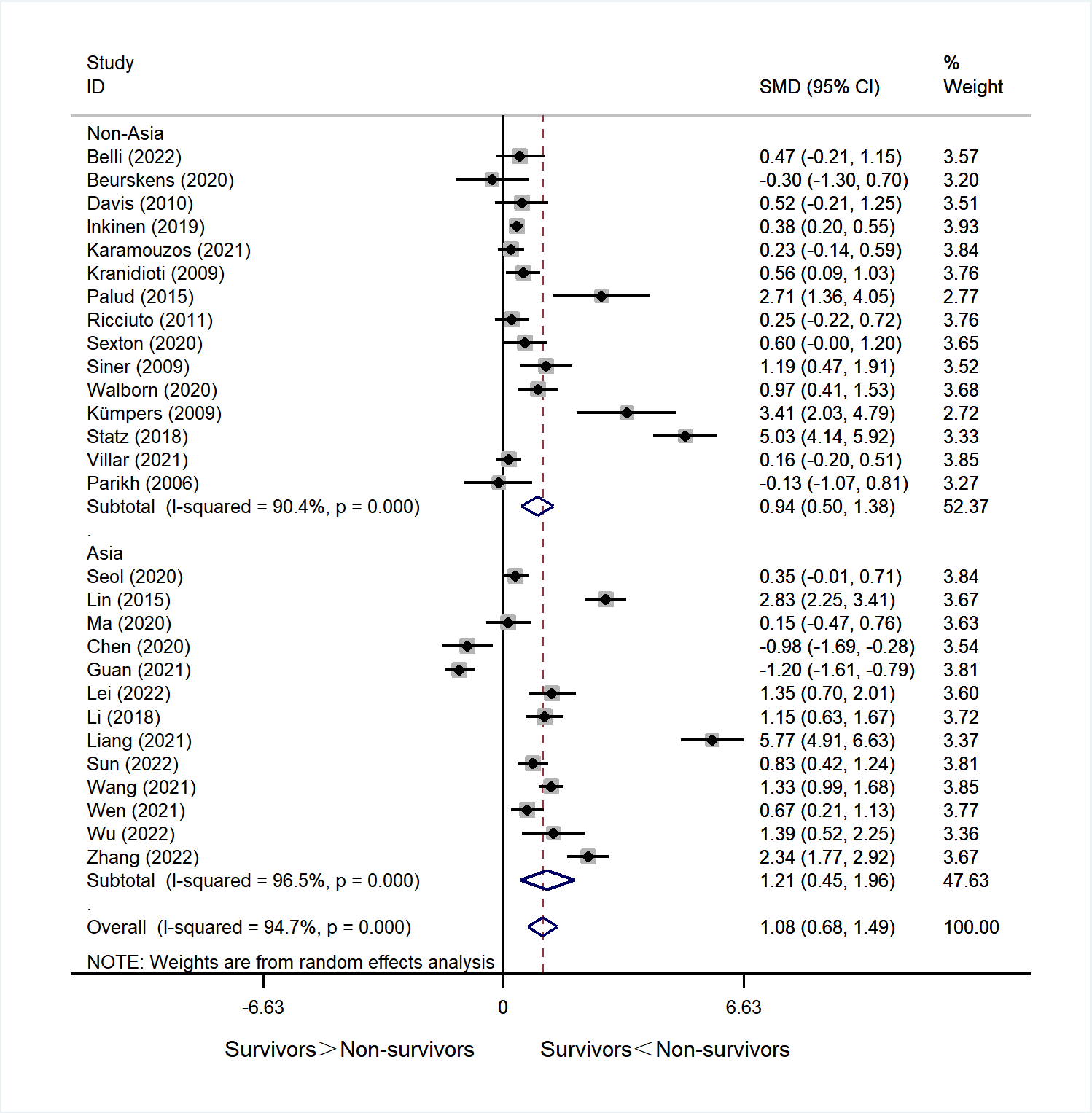


**Fig. 3** Angiopoietin-2 levels between non-survivors and survivors in septic adult patients (subgroup analysis by region)

**Abbreviations**

SMD: standardized mean differences
