# Supplementary material for: Angiopoietin-2 as a prognostic biomarker in septic adult patients: a systemic review and meta-analysis
Source: Ann Intensive Care. 2024 Nov 10;14:169. doi: 10.1186/s13613-024-01393-0 (PMC11551087; doi:10.1186/s13613-024-01393-0)
Supplement: Supplementary file 6 — Supplementary Material 6: Funnel plot and Sensitivity analysis. [file 13613_2024_1393_MOESM6_ESM.docx]

[Comparison of angiopoietin-2 Levels in Survivors Versus Non-Survivors 2](#_Toc65)

[Fig. 1 Funnel plot 2](#_Toc17181)

[Fig. 2 Filled funnel plot 3](#_Toc23887)

[Fig. 3 Sensitivity analysis 4](#_Toc921)

[Evaluation of angiopoietin-2 as a risk factor for mortality 5](#_Toc6795)

[Fig. 4 Funnel plot 5](#_Toc16111)

[Fig. 5 Filled funnel plot 6](#_Toc16901)

[Fig. 6 Sensitivity analysis 6](#_Toc9263)

[Abbreviations 7](#_Toc18143)

**Comparison of angiopoietin-2 Levels in Survivors Versus Non-Survivors**

- **Fig. 1** Funnel plot
- **Fig. 2** Filled funnel plot
- **Fig. 3** Sensitivity analysis


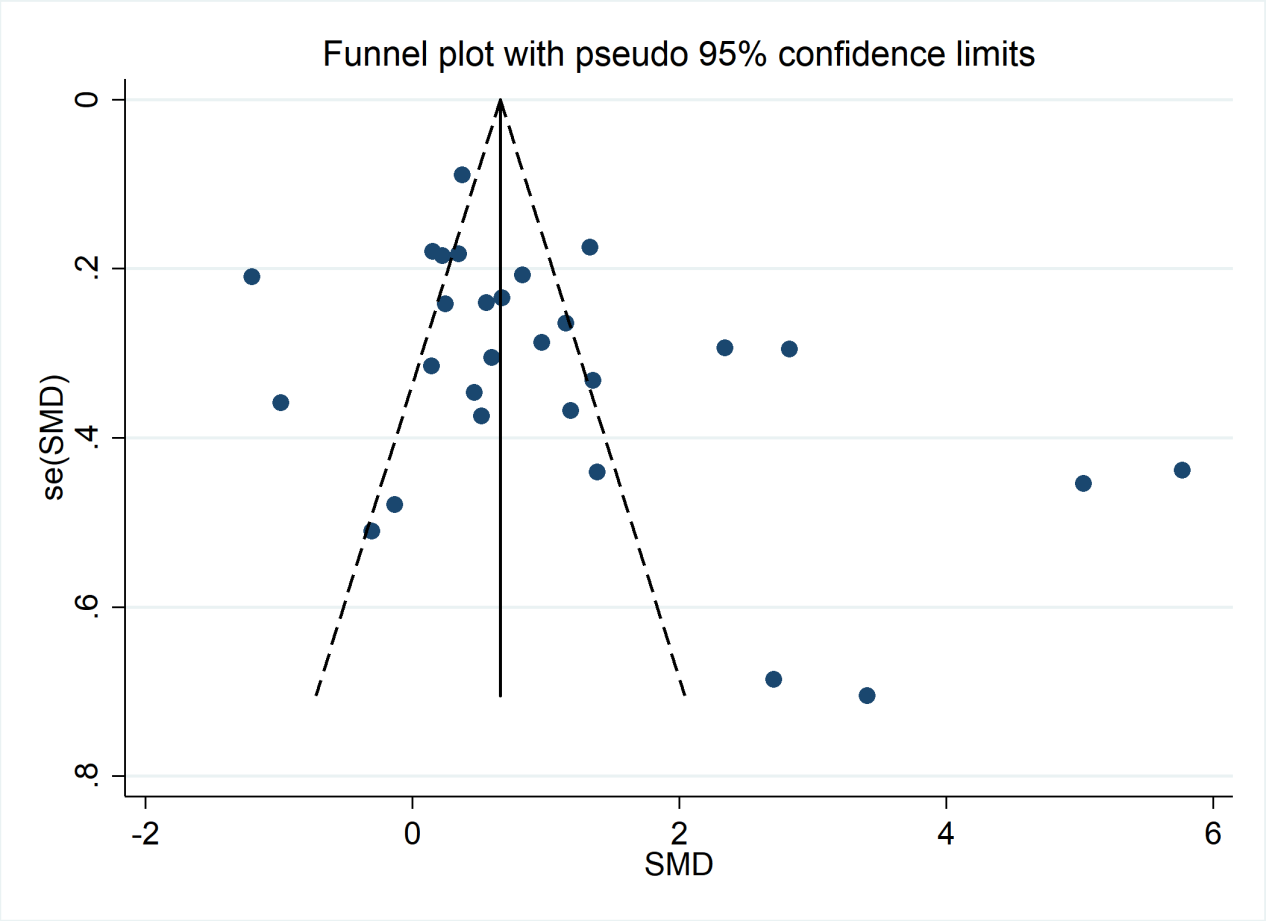


**Fig. 1** Funnel plot


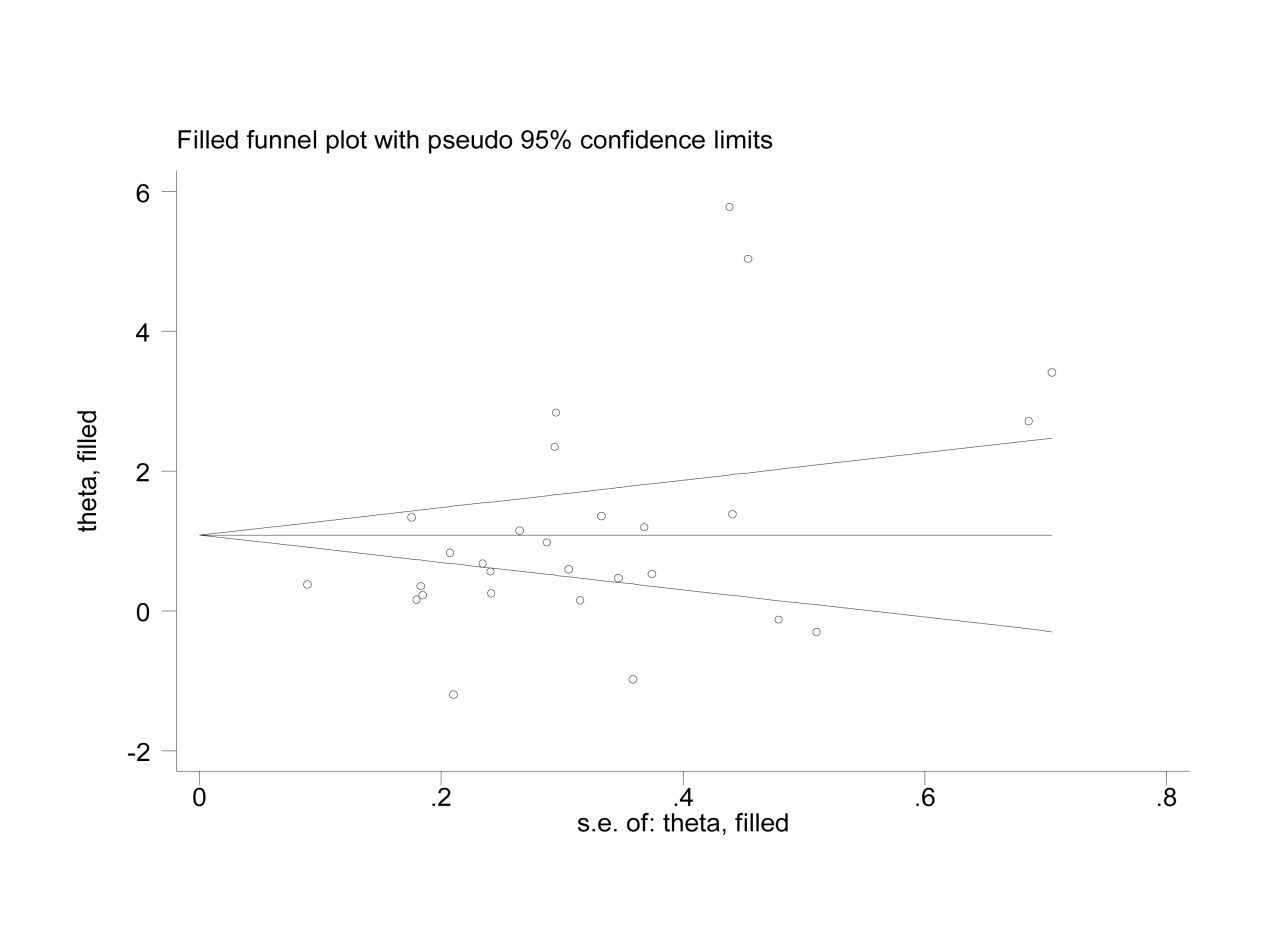


**Fig. 2** Filled funnel plot

**Fig. 3** Sensitivity analysis

**Evaluation of angiopoietin-2 as a risk factor for mortality**

- **Fig. 4** Funnel plot
- **Fig. 5** Filled funnel plot
- **Fig. 6** Sensitivity analysis


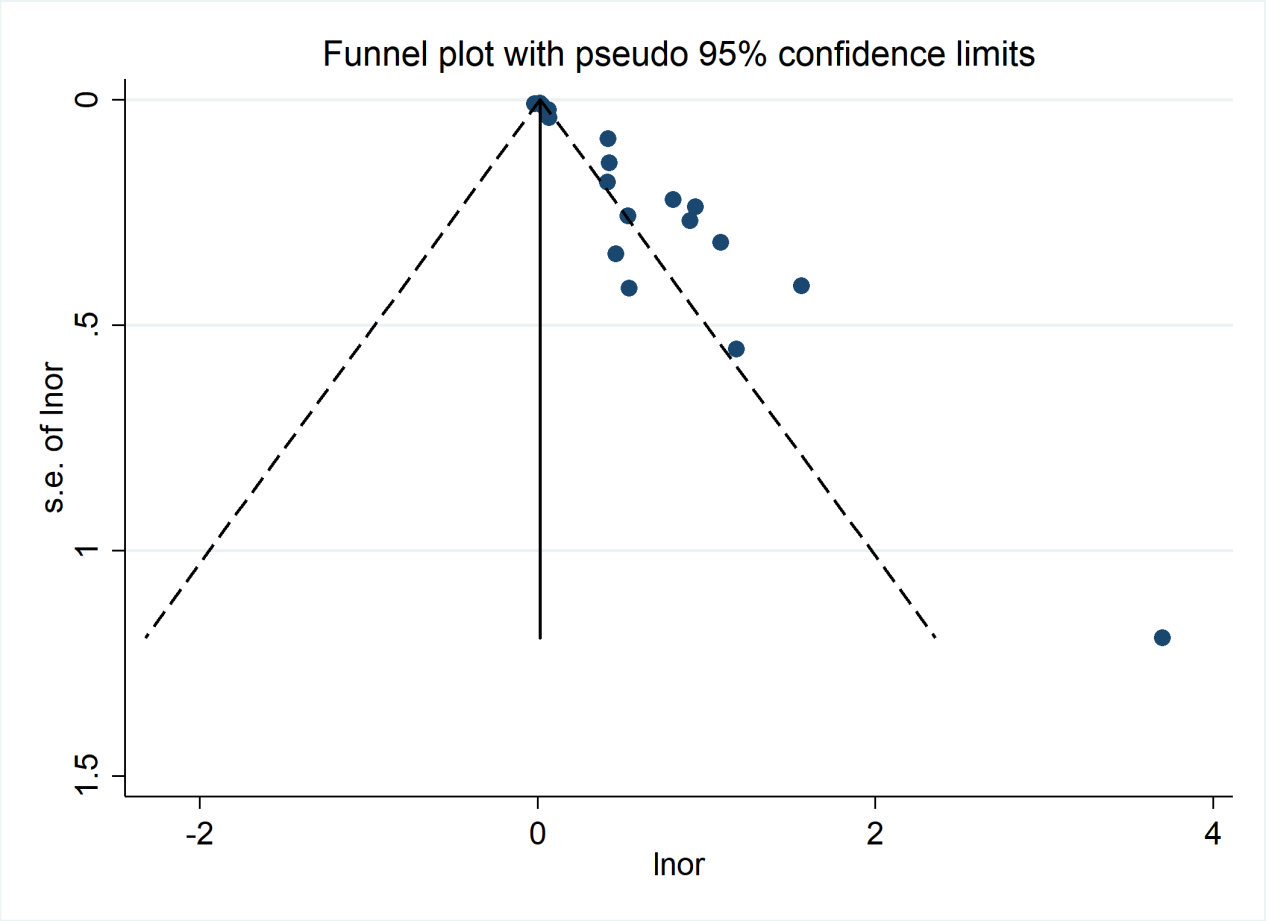


**Fig. 4** Funnel plot

**Fig. 5** Filled funnel plot

**Fig. 6** Sensitivity analysis

**Abbreviations**

SMD: standardized mean differences

CI: confidence intervals
